# Dosing and Safety of Once-Weekly Rifapentine and Isoniazid for 12 Weeks for Tuberculosis Preventive Therapy in Children With and Without HIV: Protocol for a Phase I/II Open-Label, Exposure-Controlled, Dose-Finding, and Safety Study (Tuberculosis Trials Consortium Study 35)

**DOI:** 10.2196/103274

**Published:** 2026-09-30

**Authors:** Ingrid Courtney, Meredith G Dixon, Louvina van der Laan, Rada Savic, Belen Solans, Kelly Dooley, Deron Burton, Shaun Barnabas, Avy Violari, Kia Bryant, Nigel Scott, Patrick Phillips, Erin Sizemore, Wendy Carr, Susan Purchase, Elri Nortier, Joh-Nell van der Westhuizen, Anne-Marie Demers, Anneke Hesseling, Rosanna Boyd

**Affiliations:** 1 Desmond Tutu TB Centre, Department of Paediatrics and Child Health, Faculty of Medicine and Health Sciences, Stellenbosch University, Stellenbosch, Western Cape, South Africa; 2 Centers for Disease Control and Prevention, Division of Tuberculosis Elimination, 1600 Clifton Rd NE, Atlanta, GA, 30329, United States, 1 404-553-7434; 3 Department of Bioengineering and Therapeutic Sciences, University of California, San Francisco, San Francisco, CA, United States; 4 Department of Medicine, Division of Infectious Diseases, Vanderbilt University, Nashville, TN, United States; 5 Family Centre for Research with Ubuntu (FAM-CRU), Department of Paediatrics and Child Health, Faculty of Medicine and Health Sciences, Stellenbosch University, Stellenbosch, Western Cape, South Africa; 6 Perinatal HIV Research Unit, University of the Witwatersrand, Johannesburg, Gauteng, South Africa

**Keywords:** tuberculosis, rifapentine, isoniazid, tuberculosis preventive treatment, child, HIV, pharmacokinetics, clinical trial protocol

## Abstract

**Background:**

Young children with exposure to or infection with *Mycobacterium tuberculosis* are at high risk of developing tuberculosis disease, and postexposure tuberculosis preventive treatment decreases this risk. Evidence is limited regarding the dosing and safety of the 12-dose once-weekly rifapentine and isoniazid regimen (3HP) for tuberculosis preventive treatment in children younger than 2 years and in children living with HIV.

**Objective:**

This protocol describes Tuberculosis Trials Consortium (TBTC) Study 35, which aims to establish practical age- and weight-banded rifapentine doses for 3HP in children aged 0 to 12 years, including children younger than 2 years, and children living with HIV, while assessing safety, tolerability, palatability, and acceptability.

**Methods:**

TBTC Study 35 was a phase I/II, open-label, multisite, single-arm, exposure-controlled dose-finding and safety study of 3HP in children aged 0 to 12 years, including children living with HIV. Novel water-dispersible rifapentine and isoniazid formulations were used. Building on TBTC Study 26 data, initial rifapentine doses were selected, and nonlinear mixed-effects modeling was used to analyze the pharmacokinetic data and evaluate subsequent rifapentine doses. Safety was monitored throughout the 12-week treatment period and 12-week posttreatment follow-up.

**Results:**

Data collection began with the first recruitment in November 2019. The last participant was enrolled in December 2023, and participant follow-up and data collection were completed in May 2024. As of now, 69 participants were enrolled. The trial was funded throughout this time period. Data analysis is ongoing, and trial results are expected to be disseminated in 2026.

**Conclusions:**

TBTC Study 35 is expected to provide pharmacokinetic and safety data to inform practical pediatric 3HP dosing recommendations, including for children younger than 2 years and children living with HIV.

## Introduction

### Tuberculosis in Children

The World Health Organization (WHO) estimated that 1.3 million children younger than 15 years of age developed tuberculosis (TB) in 2023 [1], with most cases being preventable. Following exposure to and infection with *Mycobacterium tuberculosis,* young children, especially those younger than 5 years of age, have a high risk of progressing to active TB disease, with the highest risk in children younger than 2 years and in those living with HIV [2,3]. Young children also have a propensity to develop severe and disseminated forms of TB disease [2,4]. TB mortality, specifically among children younger than 5 years, was estimated as 139,000 (95% UI 125,000‐154,000) in 2022, in the absence of adequate TB treatment [5].

### TB Preventive Treatment

Postexposure TB preventive treatment (TPT) is an important and effective public health strategy to reduce the burden of TB among children [2]. However, adherence to long courses such as 6 to 9 months of isoniazid preventive therapy is generally low, particularly in children living in high-burden settings [6,7], thereby limiting the practical effectiveness of this strategy. Shorter TPT regimens in children are critically important to ensure better and more equitable access to effective and child-friendly TPT regimens.

### Rifapentine for TPT

Shorter rifamycin-based regimens offer an alternative to longer courses of isoniazid for TPT. Four months of daily rifampicin (4R) is an option for TPT, although its use is limited in people living with HIV who are taking certain antiretroviral (ARV) medications because of drug-drug interactions [8]. Rifapentine is another rifamycin with a prolonged half-life (t½) that supports less frequent dosing and has a lower minimum inhibitory concentration against *M. tuberculosis* than rifampicin. It is approved for TPT. A regimen of once-weekly rifapentine plus isoniazid for 12 weeks had potent activity in an animal model of latent TB infection [9]. A large-phase 3 trial comparing this regimen with 9 months of daily isoniazid, Tuberculosis Trials Consortium (TBTC) Study 26 (n=8053, “PREVENT TB”; NCT00023452), found that the 12-week combined rifapentine and isoniazid regimen (3HP) given by directly observed therapy was at least as effective as self-administered isoniazid taken daily for 9 months in preventing TB disease [10,11].

In a pediatric study nested within TBTC Study 26, the efficacy and safety of 3HP were assessed in children aged 2 to 17 years. The regimen was safe and at least as effective as 9 months of daily isoniazid [10,11]. It also had a higher treatment completion rate than the isoniazid-only group [10,11].

### Pharmacokinetics of Rifapentine in Children

In TBTC Study 26 pharmacokinetics (PK) substudy (NCT00164450), rifapentine PK was evaluated in children aged 2 to <12 years and compared with that in adults [12,13]. Children received weight-based rifapentine with once-weekly isoniazid. Adult 150-mg film-coated rifapentine tablets (Priftin, Sanofi) were crushed for children who could not swallow whole tablets. Children received higher rifapentine doses in mg/kg than adults, and these doses were well-tolerated. However, children receiving crushed tablets had lower rifapentine exposure than those who swallowed whole tablets [13]. Children younger than 2 years and children living with HIV were excluded from TBTC Study 26 and the PK substudy, given the lack of a child-friendly rifapentine formulation at the time, resulting in limited guidelines and access for 3HP dosing in these younger children. This evidence gap was particularly important in young children, including those younger than 2 years of age, due to developmental changes in body composition, organ function, and drug-metabolizing pathways, which may alter drug disposition, limiting direct extrapolation of PK data from adults and older children to young children. Novel water-dispersible rifapentine formulations were subsequently developed by the drug originator (Sanofi) for evaluation in children.

Building on the efficacy and safety evidence available for children aged 2 years and older, TBTC Study 35 aimed to provide PK and safety data, particularly for children younger than 2 years, to guide optimal dosing of 3HP in children across the age spectrum, including those living with HIV.

TBTC Study 35 aimed to derive rifapentine doses expected to achieve adult-equivalent exposures when given in combination with isoniazid through modeling existing PK data from Study 26 [12,13], together with interim Study 35 PK data collected after predefined cohort evaluations [10,13,14]. The trial also evaluated the safety, tolerability, and palatability of 3HP using novel water-dispersible formulations in children. In addition, including children living with HIV receiving appropriate ARV regimens, such as dolutegravir-based ARV treatment, was important because there were limited data on rifapentine and ARV drug-drug interactions and on rifapentine PK in children. These data will support the evaluation of appropriate once-weekly rifapentine dosing for TPT in children living with HIV [15].

## Methods

### Overall Design and Objectives

TBTC Study 35 was a phase I/II, open-label, multisite, single-arm, exposure-controlled dose-finding study designed to evaluate the PK, safety, tolerability, and palatability of water-dispersible formulations of rifapentine given in combination with isoniazid as TPT (3HP) in children at high risk of developing TB. The study used a modified age de-escalation design, with participants enrolled in 4 age cohorts: cohort 1 (≥4 to ≤12 y), cohort 2 (≥2 to <4 y), cohort 3 (≥1 to <2 y), and cohort 4 (0 to <1 y). The total targeted enrollment was a minimum of 12 participants each in cohorts 1 and 2 and 18 participants each in cohorts 3 and 4, respectively, for a minimum total evaluable number of 60; there was no formal enrollment target for children living with HIV. Figure 1 (study schematic) summarized enrollment, PK, and safety assessments, interim analyses, and decision criteria for rifapentine dose adjustments. Cohorts 1 and 2 were enrolled in parallel, followed by an enrollment pause and interim PK/safety analysis after at least 6 participants were enrolled in each cohort and had available data. The median area under the plasma concentration-time curve (AUC) of rifapentine was compared to that of adults in TBTC Study 26 [10,11]. The enrollment of cohorts 1 and 2 was then continued, and cohorts 3 and 4 were added and enrolled in parallel, followed by a second enrollment pause and interim analysis after at least 6 participants were enrolled in each of cohorts 3 and 4 and had available data. Dose adjustments were implemented after each interim analysis if there was a deviation from the estimated target exposure and/or if safety targets were not met. The final age- and weight-banded rifapentine dosing algorithm was selected using population PK simulations and with consideration of the commercial formulations available for practical dosing. Candidate dose bands were evaluated against the prespecified adult rifapentine AUC target range, cohort-level safety findings, and available tablet strengths. Weight was incorporated through allometric scaling, while age, HIV status, antiretroviral therapy (ART) regimen, and other prespecified covariates were evaluated in the population PK model and retained when supported by the data and clinically relevant. Final dose selection favored the simplest practical regimen that achieved adult target exposure across age and weight groups without exceeding protocol-defined safety thresholds.

**Figure 1.**
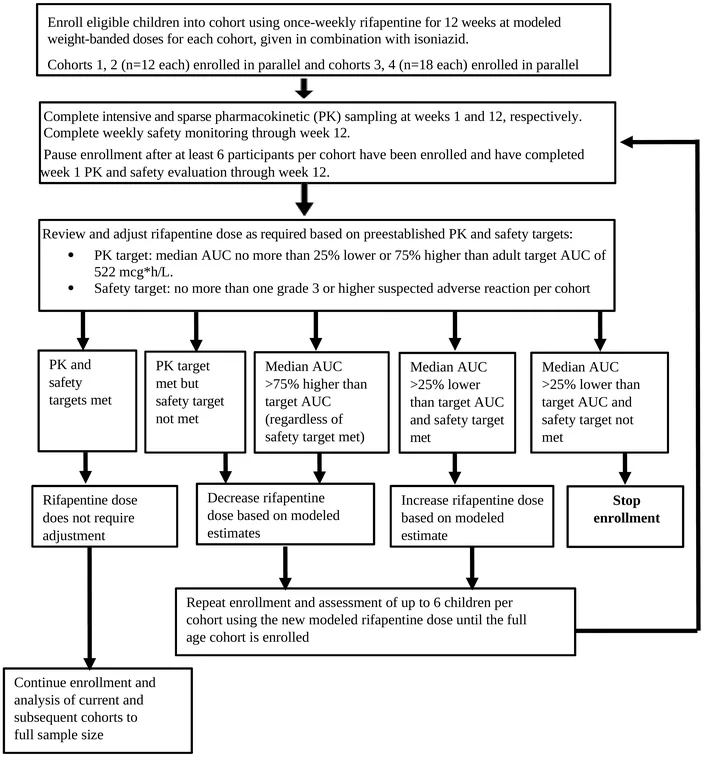
Cohort overview and management of age cohorts in children in TBTC Study 35. AUC: area under the plasma concentration-time curve.

The primary objective was to establish, through population PK modeling, a dosing algorithm derived by the simulation of rifapentine doses that would achieve the target adult exposure from Study 26 [10,11]. The secondary objectives were to document the safety, tolerability, palatability, and acceptability of the study regimen; to characterize the PK of rifapentine and isoniazid, taking into account covariates that may influence the PK characteristics, including age, weight, sex, HIV status, nutritional status, and pharmacogenomics; and to characterize incident TB over 24-week follow-up from enrollment. Exploratory objectives were to characterize the effects of 3HP on efavirenz in children living with HIV, accounting for CYP2B6 efavirenz metabolizer status, and to investigate the drug-drug interaction between integrase inhibitors (eg, dolutegravir) and rifapentine. Because no formal enrollment target was set for children living with HIV or individual ART regimens, estimates from small HIV/ART subgroups were considered exploratory, presented with uncertainty, and interpreted descriptively rather than as definitive regimen-specific dosing conclusions.

### Study Setting and Recruitment

TBTC Study 35 was conducted in South Africa, a setting with high burden of TB and HIV, at 3 research sites (Desmond Tutu TB Centre and Family Centre for Research with Ubuntu, Stellenbosch University, and the Perinatal HIV Research Unit, Wits Health Consortium, Johannesburg). Recruitment occurred through referrals from community-based clinics or hospitals where children were routinely identified through TB contact investigation and referred for management or from local HIV treatment clinics. Written informed consent and written assent, as appropriate (children ≥7 years of age), in the preferred home language of the parent/legal guardian and the child as appropriate (English, Afrikaans, and isiXhosa) were obtained before any study-specific procedures were performed. Additional consent was obtained for pharmacogenetic testing and future use of samples.

### Study Population and Eligibility

Children aged 0 to 12 years, with or without HIV, who had documented recent close exposure to an adult with bacteriologically confirmed, drug-susceptible pulmonary TB or who had a positive test for *M. tuberculosis* infection were eligible. All participants were screened for TB infection and disease at baseline prior to enrollment. Screening included the following: (1) a Mantoux tuberculin skin test (TST; TB infection defined as ≥10 mm in children living without HIV and ≥5 mm in children living with HIV) or QuantiFERON-TB Gold Plus (Qiagen); (2) symptom screening, clinical assessment, and anthropometry; (3) chest radiographs (anteroposterior and lateral); and (4) bacteriological investigations, including 2 high-quality respiratory or other samples for molecular testing and mycobacterial culture and drug susceptibility testing (Global Central Laboratory BARC).

Inclusion and exclusion criteria are listed in Textbox 1. Children living with HIV were established on ART with efavirenz-, dolutegravir-, or raltegravir-based regimens for at least 12 weeks prior to enrollment. Exclusion criteria included active TB disease, documented drug-resistant TB exposure, TB treatment in the preceding 30 days, hemoglobin <9 g/dL, weight-for-age *z* score <−2, known hypersensitivity to isoniazid or rifapentine, weight <2.5 kg and >40 kg, hepatic dysfunction (>5- fold elevated alanine aminotransferase and/or bilirubin), Lansky play score <50, or active hepatitis A/B infection. Female adolescents who had reached menarche were excluded to ensure that no pregnant individuals were treated in this trial.

Textbox 1. **Participant eligibility criteria for Tuberculosis Trials Consortium Study 35.**

Enrollment will be *deferred,* as clinically appropriate, in HIV-positive and HIV-negative participants with acute severe illness including severe respiratory infection, diarrheal disease, acute central nervous system, febrile or other acute illness, or any other relevant clinical condition, as judged by the clinical investigator. Participants may then be rescreened for study eligibility. If children have new TB exposure reported during the study, they should be screened again for TB as clinically indicated.

**Inclusion criteria**

Individuals must meet all of the following inclusion criteria in order to participate in this study:

- Aged 0 to 12 years
- Documented close (household or other) exposure (for at least an average of 4 hours a day that occurred in the preceding 6 months before study enrollment to a bacteriologically confirmed source case with pulmonary TB. The exposure can be of any duration, that is, weeks or months, but it must be exposure that occurs for more than 4 hours a day during the 6 months preceding study enrollment. The TB source case should have confirmed drug-sensitive (sputum culture confirmed or XPERT MTB/Rif or other WHO-approved molecular test[s] TB and without any evidence of drug resistance, that is, at least XPERT MTB/Rif or Xpert MTB/RIF Ultra rifampicin susceptible or an alternative molecular, for example, line probe assay or phenotypic test indicating rifampicin susceptible *M. tuberculosis*). TB infection included those with positive tuberculin skin test (TST) ≥10 mm in HIV-negative and TST ≥5 mm in HIV-positive participants or a positive commercial interferon-gamma release assay, as defined by the manufacturer.) OR Evidence of *M. tuberculosis* infection
- Confirmed HIV status (confirmed by DNA polymerase chain reaction or plasma HIV-RNA if participant is <18 months of age, or ≥18 months and with any exposure to breast milk for at least 28 days prior to the time of HIV testing. In participants ≥18 months of age, HIV ELISA (enzyme-linked immunosorbent assay) testing will be completed. If any HIV test is positive in a child participant, regardless of age, the test result needs to be confirmed with a second HIV test, using HIV DNA or RNA polymerase chain reaction, from an independent sample, that is, 2 independent documented positive tests.)
- HIV-positive participants should be on an ART regimen for at least 12 weeks prior to enrollment and should be clinically stable before entering the study, regardless of CD4 (cluster of differentiation 4) lymphocyte count and HIV viral load. While on study, participants must be on an efavirenz- or dolutegravir-based or raltegravir-based ART regimen, which should have been given for at least 14 days prior to enrollment
- Caregiver (parent or legal guardian) gives written informed consent and assent from the child where applicable
- Weight >2.5 kg but <40 kg

**Exclusion criteria**

An individual meeting any of the following exclusion criteria will be excluded from study participation:

- Active TB disease (evidenced by: symptoms suggestive of TB or suggestive findings on clinical examination or suggestive chest radiographic findings or positive mycobacterial culture/molecular TB tests if culture/molecular testing was clinically indicated and was completed, or currently on TB treatment for active disease)
- Any documented drug-resistant TB in an identified infectious source case (defined as genetic mutations associated with rifampicin resistance detected on Xpert or any other relevant approved molecular test or phenotypic evidence of rifampicin resistance)
- Total receipt of >30 days of a once-daily isoniazid regimen AND given for at least 14 consecutive days during the 30 days prior to enrollment
- Hemoglobin <9 g/dL
- Weight for age z score below −2 or severe clinical malnutrition
- Known allergies or hypersensitivity to isoniazid or rifapentine
- Documented hepatic disorder including >5-fold elevated upper limit of normal alanine aminotransferase and/or bilirubin
- Lansky play score <50
- Documentation of active current hepatitis A or B infection
- Female adolescents who have reached menarche will not be eligible

### Study Treatment, Formulations, and Dosing

Participants received 3HP using water-dispersible fixed-dose combination tablets (150 mg rifapentine/150 mg isoniazid, Sanofi) and stand-alone 100 mg of water-dispersible rifapentine tablets (Sanofi), if a rifapentine top-up was required. Both layers of rifapentine and isoniazid in the fixed-dose combination were homothetic to the stand-alone formulations of rifapentine and isoniazid (having the same qualitative and quantitative composition and the same manufacturing process, differing only in masses). When tablets were divided, the portions were weighed to ensure accurate dosing, and the weights were documented. Tablets were dispersed in 3 to 20 milliliter of water, with a small-volume rinse (1‐5 mL). Doses were administered under direct observation 30 to 60 minutes after an age-appropriate, high-fat meal was supplied by the study team. The meal and the quantity consumed were documented.

Cohorts 1 and 2 started at an approximate rifapentine dose of 20 to 25 mg/kg (targeted to achieve adult-equivalent exposure) and an isoniazid dose of approximately 25 mg/kg. The approximately 25 mg/kg isoniazid target was implemented through study weight bands, resulting in an administered range of 15 to 25 mg/kg. Weight at enrollment was used to determine dosing during the 12 weeks of study treatment (ie, no weight adjustment was made over 12 weeks). The study design enabled rifapentine dose adjustments for cohorts, but not for individual study participants, given the lack of real-time assay data. Isoniazid doses were weight based (15‐25 mg/kg) with no interim analyses or dose adjustments, as pediatric isoniazid PK were well established [16,17]. Children younger than 4 years received pyridoxine 12.5 mg once weekly, and those aged ≥4 years received 25 mg once weekly with 3HP.

### Participant Management

Participants were followed for 24 weeks, including 12 weeks on 3HP (“study treatment” period) and 12 weeks post treatment (“follow-up” period) to monitor for incident TB. At screening, participants underwent assessment for HIV status, medical and TB exposure history, complete physical examination and anthropometry, TST or commercial interferon-gamma release assay, chest X-ray, hepatitis studies, hematology (complete blood count with differential), selected chemistry (sodium, potassium, urea, and creatinine), and selected liver function tests (alanine aminotransferase, total bilirubin, and albumin). For children living with HIV, CD4 (cluster of differentiation 4) lymphocyte count and viral load were assessed at screening and again at 24 weeks. Adverse event (AE) assessments were performed at every study visit and during follow-up visits, as clinically indicated. The schedule of study evaluations is included in Table 1 including the timing of intensive and sparse PK sampling.

**Table 1.** Schedule of study evaluations in Tuberculosis Trials Consortium Study 35.

Study evaluations	Screening	Week 1	Week 2	Week 3	Week 4	Week 5	Week 6	Week 7	Week 8	Week 9	Week 10	Week 11	Week 12	Week 16	Week 20	Week 24	Early study/drug discontinuation
Clinical evaluations																	
Informed consent/assent	✓																
Demographic data	✓																
Tuberculosis contact history	✓																
HIV status (history)	✓																
Medical history (including adverse event reporting)	✓	✓	✓	✓	✓	✓	✓	✓	✓	✓	✓	✓	✓	✓	✓	✓	✓
Complete physical exam (includes vital signs)	✓												✓		✓		✓
Targeted physical exam (includes vital signs)		✓	✓		✓		✓		✓		✓			✓		✓	
Anthropometry (weight, length, and MUAC^a^)	✓	✓	✓		✓		✓		✓		✓		✓	✓		✓	
Study drug dispensing and directly observed treatment		✓	✓	✓	✓	✓	✓	✓	✓	✓	✓	✓	✓				
Adherence assessment (concomitant medications)		✓	✓	✓	✓	✓	✓	✓	✓	✓	✓	✓	✓	✓	✓	✓	✓
Palatability and acceptability		✓					✓						✓				
Tuberculin skin testing or interferon-gamma release assay	4.0 mL^b^						✓										
Chest radiography	✓																
Laboratory evaluations																	
HIV testing	1.0 mL															1.0 mL	
Hepatitis serology (A, B)	1.0 mL																
PK^c^ (intensive sampling)		3.0 mL															
PK (sparse sampling)													1.0 mL			1.0 mL	
Pharmacogenetic testing		1.0 mL															
Hematology (CBC^d^, differential)	0.5 mL		0.5 mL		0.5 mL				0.5 mL				0.5 mL				0.5 mL
Urea, creatinine, sodium, and potassium	0.5 mL		0.5 mL		0.5 mL				0.5 mL				0.5 mL				0.5 mL
Liver function tests (ALT^e^, bilirubin), albumin	0.5 mL		0.5 mL		0.5 mL				0.5 mL				0.5 mL				0.5 mL
HIV+ participants only																	
WHO^f^ HIV staging	✓																
ARV^g^ adherence	✓	✓	✓	✓	✓	✓	✓	✓	✓	✓	✓	✓	✓	✓	✓	✓	✓
CD4^h^ lymphocyte count	1.0 mL															1.0 mL	
HIV viral load	1.0 mL															1.0 mL	
ARV PK sampling		1.0 mL											1.0 mL			1.0 mL	

a MUAC: mid-upper arm circumference.

b mL: milliliters.

c PK: pharmacokinetics.

d CBC: complete blood count.

e ALT: alanine aminotransferase.

f WHO: World Health Organization.

g ARV: antiretroviral.

h CD4: cluster of differentiation 4.

### Palatability and Acceptability Assessments

Palatability and acceptability were assessed repeatedly during treatment using a standard validated Five-point Facial Hedonic Scale questionnaire completed by participants when age appropriate or by caregivers for younger children. At selected sites, an in-depth qualitative substudy explored the acceptability of and experiences with 3HP among children, caregivers, and health care workers. These methods and findings will be reported separately.

### PK Sampling

The PK sampling strategy was informed by nonlinear mixed effects modeling and prior data from adults, adolescents, and children from the Study 26 PK substudy [12,13]. At week 1, participants underwent intensive PK sampling with 6 venous blood samples collected predose (t0) and at 1, 2, 4, 8, and 24 hours post dose based on the long observed half-life of rifapentine (t ½ 17 h). Sampling followed directly observed drug administration with food provided by the study team. At approximately week 12, participants underwent sparse PK sampling, with 2 venous blood samples collected at 1 and 24 hours post dose. The exact PK sampling times were documented.

For children living with HIV, the previous evening’s and the morning’s ARV doses were documented at the PK visits. Children living with HIV served as their own controls with repeated PK sampling 12 weeks after completion of rifapentine and isoniazid (ie, at approximately week 24), using semi-intensive sampling (0, 1, and 6 h post ARV dose) to assess potential drug-drug interactions between rifapentine and ARVs. Minimal volume assays (<100 μL per assay) were performed using a modified version of a published validated liquid chromatography-tandem mass spectrometry assay at the University of Cape Town [18].

### Pharmacogenetic Sampling

Pharmacogenetic samples were collected at week 1 and stored as buffy coat samples before central analysis. Genetic testing was optional and limited to participants who provided consent. Testing investigated the effects of prespecified genotypes, including SLCO1B1 and NAT2 on the PK of rifapentine, isoniazid, and relevant ARVs.

### PK and Safety Targets

The rifapentine PK target assessed at each interim analysis was an AUC no more than 25% lower or 75% higher than the target AUC of 522 mg·h/L [10,13]. This corresponded to an acceptable AUC range of approximately 392 to 914 mg·h/L. In historical pediatric controls, the typical rifapentine clearance (CL) was 1.83 L/h for a 35.0 kg child, with CL allometrically scaled as CL=1.83 L/h×(WT/35 kg)^0.75^; where is WT is body weight) and a maturation function applied as described by Weiner et al [13] and Savic et al [19]. Safety targets for each interim analysis required that no more than one grade 3 or higher AE possibly or definitely related to a study drug (rifapentine or isoniazid) occur in any cohort. Dose adjustment was implemented after each interim analysis if there was a deviation from the estimated target exposure and/or if safety targets were not met.

An independent Data and Safety Monitoring Board (DSMB) was composed of a clinical TB expert, an epidemiologist, and a clinical statistician, all of whom were not otherwise involved in the study. The DSMB convened approximately annually, or more frequently if required, to review study progress and safety data. The core study team conducted the prespecified interim PK and safety analyses and reported the results and any proposed dose adjustments to the independent DSMB for review. The independent DSMB reviewed protocol-defined interim analyses and safety data, advised on dose adjustments, and could recommend early study termination. Enrollment remained paused while interim modeling and safety review were completed; no fixed implementation interval was prespecified.

### Sample Size Assumptions

Sample size estimates were based on the primary objective of establishing a dosing algorithm through population PK modeling and were informed by (1) the comparison of pediatric PK parameters with those of adults receiving 900 mg of rifapentine (the effective dose for TPT in TBTC Study 26 [10,11]) and (2) prior PK data from TBTC Study 25 [20]. This led to a sample size of approximately 60 participants with enrichment in the younger cohorts. The stochastic simulation-estimation methodology for clinical trial simulation [21] was used to assess the sample size and the number of blood samples required to estimate key PK parameters with sufficient precision for decision-making. Based on this power analysis, 12 participants in each of cohorts 1 and 2 and 18 participants in each of cohorts 3 and 4 (Table 2), with 6 intensive PK samples obtained at week 1 (predose and 1, 2, 4, 8, and 24 hours post dose), followed by sparse sampling (1 and 24 h post dose) at approximately week 12, were considered sufficient. A percentage coefficient of variation (%CV) of 50% was assumed, based on existing C24 data in participants [22]. Formal power analysis indicated at least 80% power to detect a >30% change in a PK parameter (50%CV) between 2 groups of 12 participants; power increased when week 1 and approximately week 12 data were combined. For the younger age cohorts, 18 participants per cohort were included to account for potentially higher variability in PK parameters in children younger than 2 years. When combined in a single model, the power to detect differences in PK parameters between children and adults was expected to be even greater. A retention rate of at least 90% was assumed, based on retention data from ongoing pediatric studies, including household-contact studies with active screening and follow-up.

**Table 2.** Sample size estimates by relevant age strata.

Cohort	Age range	Total number
4	0 to 12 mo	18
3	13 to 24 mo	18
2	25 mo to 4 y	12
1	4 to 12 y	12
Total		60

### Analysis Plan

Population PK analysis will characterize the PK profiles of rifapentine, isoniazid, and ARVs in children using a compartmental modeling approach. The model structure is flexible, potentially incorporating one or more compartments for absorption and disposition kinetics, to be determined based on the data and drug characteristics. Primary parameters estimated include the absorption rate constant (ka), CL, and volume of distribution. Allometric scaling will be applied to account for body size differences, with weight as a key predictor of CL and volume of distribution, potentially supplemented by age or nutritional status to capture developmental changes [23-25]. Alternative descriptors of body size, such as fat-free mass, will also be evaluated [26]. Model development will incorporate linear or nonlinear age-dependent functions to describe longitudinal changes in PK parameters. Model building and evaluation will use standard diagnostics, including visual predictive checks and bootstrap methods, to support robust parameter estimation [27]. Covariate effects will be assessed using stepwise covariate modeling, with significance thresholds of *P*<.05 for inclusion and *P*<.01 for retention. Model development and estimation will be conducted in NONMEM (ICON Development Solutions), using first-order conditional estimation with interaction.

Safety-related end points are the cumulative number and proportion of children with grade ≥3 AEs, and the cumulative number who discontinue the study drug due to an AE, over the 12-week dosing period. In addition to capturing all AEs, reportable events to the sponsor and regulatory bodies included any grade 2 or higher sign, symptom, or laboratory value; a new diagnosis with any grade ≥2 component; an AE leading to treatment discontinuation; worsening of a preexisting diagnosis to grade ≥2; a clinically significant laboratory result; or an AE of special interest. AE of special interest included rifamycin hypersensitivity syndrome and hepatotoxicity (symptomatic hepatitis). The Division of AIDS Table for Grading the Severity of Adult and Pediatric Adverse Events, version 2.1, dated July 2017, was used to assess AE severity. Relatedness to the study drugs was assessed (attribution as possible, probable, or definite was classified as related). AEs were reviewed in near-real time by a central study physician and coded using the Medical Dictionary for Regulatory Activities (MedDRA) version 20.0.

For interim safety analyses, the threshold for dose-limiting toxicity (DLT) was set at 2 or more grade 3 or higher suspected adverse reactions associated with at least one study drug (rifapentine or isoniazid) among the first 6 participants enrolled in a given cohort at a given rifapentine dose. If DLT occurred in 2 or more of 6 participants within an interim-analysis age-dose cohort, the dose received by these participants was considered unacceptably high for the respective age cohort. Study-drug doses equal to or greater than a toxic dose level were not to be used for additional participants enrolled in the respective age cohort. The core study team conducted interim PK and safety analyses. The independent DSMB reviewed interim analyses and safety data, advised on dose adjustments, and could recommend early termination. Enrollment remained paused while interim modeling and safety review were completed and no fixed implementation interval was prespecified. In addition to the DLT rule, the prespecified cohort safety target required no more than one grade ≥3 AE assessed as possibly or definitely related to rifapentine or isoniazid in each cohort. Overall safety analyses will report the number and proportion of participants who experience: any AE, any AE of grade 3 or higher, any suspected adverse reaction, any suspected adverse reaction of grade 3 or higher, or any serious AE. Descriptive tables will list each suspected adverse reaction and key clinical details.

For overall safety analyses, the observed proportion of participants with grade 3 or higher suspected adverse reactions will be compared with the prespecified reference proportion of 5%, based on pediatric data from TBTC Study 26 [10,11]. The study investigators determined that safety during 12 weeks of study treatment and follow-up would be considered acceptable if no more than one participant per cohort had a grade ≥3 AE related to study treatment, and the lower bound of the 95% Clopper-Pearson CI of the overall proportion of such AEs was not larger than 5%.

Thus, the null and alternative hypotheses are as follows:

- *H0*: *P*=*p0* (where *p0* is defined as 0.05)
- *H1*: *P*=*p1* (where *p1*>*p0*)

If 60 evaluable participants are enrolled, 7 grade ≥3 suspected adverse reactions would allow for the rejection of the null hypothesis (nominal type 1 error rate of 0.05; nominal power 0.8). The type 1 error rate associated with this critical value for adverse safety events is 0.03. Power is 70% to detect a true adverse safety event rate of 0.13 (8 out of 60 participants), 82% for 0.15 (9 out of 60), and 89% for 0.17 (10 out of 60).

### Ethical Considerations

The trial protocol was approved by the Centers for Disease Control and Prevention Institutional Review Board, the South African Health Products Regulatory Authority, the Stellenbosch University Human Research Ethics Committee, the University of the Witwatersrand Human Research Ethics Committee, and the WHO Ethics Review Committee. The study was conducted under a US Food and Drug Administration Investigational New Drug application (141932). Written informed consent from a parent or legal guardian and age-appropriate child assent were obtained before study-specific procedures were performed.

### Dissemination of Trial Findings

Data from this trial are intended to inform updated WHO and other clinical/public health guidelines for dosing 3HP in children across all weight bands and in children living with HIV, enabling broader access to 3HP. The results will be shared with WHO and local community advisory boards, presented at scientific meetings and local health services meetings, and published in peer-reviewed journals in accordance with the CONSORT (Consolidated Standards of Reporting Trials) statement. Additional dissemination of results will be through the press, national meetings, professional bodies, and international conferences.

## Results

Data collection began with the first recruitment in November 2019. The last participant was enrolled in December 2023, and participant follow-up and data collection were completed in May 2024. As of now, 69 participants have been enrolled. The trial was funded throughout this time period. Data analysis is ongoing, and trial results are expected to be disseminated in 2026.

## Discussion

### Addressing Evidence Gaps

The WHO first recommended 3HP as an alternative to the 6 months of daily isoniazid (6H) in 2018 [28]. The recommendation applied to adults and children aged 2 years and older, regardless of HIV status or TB burden setting [28]. The rationale was based on evidence showing that 3HP had similar efficacy to 6H, but with improved treatment completion rates and fewer adverse effects, including hepatotoxicity [10]. However, the PK characteristics of rifapentine given as water-dispersible formulations had not previously been evaluated in children. The study of rifapentine in this new formulation is important especially in children younger than 2 years, who are at the highest risk of TB, and for whom drug administration can be challenging. Study 35 will provide data for dosing recommendations for TB prevention in children in order to safely achieve target adult rifapentine exposures that are correlated with efficacy and safety [10,11], including in children living with HIV on appropriate ARV regimens [2,3].

Data regarding the safety and tolerability of rifapentine in young children are critically important, given the high risk of TB disease and the existing clear evidence regarding the efficacy and safety of the 3HP regimen in adults and older children [9,10]. Shorter duration, intermittently dosed combination TPT may be an effective and feasible strategy to improve adherence and thus clinical outcomes in children compared to 6H recommended in international settings.

The DOLPHIN trial evaluated 3HP for TB prevention in 60 adults living with HIV receiving a dolutegravir-based ART regimen [29]. Although 3HP reduced dolutegravir trough concentrations, the geometric mean trough concentration remained above the target, and HIV viral suppression was maintained in all participants [15]. The regimen was safe and well-tolerated [15,29]. The study concluded that rifapentine and isoniazid could be administered for TPT without dolutegravir dose adjustment in adults living with HIV receiving dolutegravir-based ART [15,29]. Given the potential for drug-drug interactions between rifapentine and ARV drugs in children, and the burden of TB in children living with HIV, we anticipate that TBTC Study 35 will provide important pediatric PK and safety data on the coadministration of dolutegravir and 3HP. These findings will help guide the global use of 3HP in young children and children living with HIV and may significantly impact TB prevention in children.

### Limitations

The single-arm, exposure-controlled design is appropriate for dose finding but limits direct comparisons of clinical effectiveness. The study was conducted at South African sites, which may limit generalizability to other epidemiologic settings, ARV regimens, and nutritional profiles. Other factors, such as genetic variability in drug disposition, may have been limited because participants were enrolled in a single country, despite considerable genetic heterogeneity across the 3 sites. The target sample size is sufficient for population PK modeling but may not detect rare AEs.

### Conclusions

TBTC Study 35 is expected to provide the PK and safety evidence needed to inform practical pediatric 3HP dosing recommendations and to support access for children younger than 2 years and for children living with HIV.

## Abbreviations

3HP: once-weekly regimen of rifapentine and isoniazid for 12 weeks for tuberculosis prevention
6H: 6 months of daily isoniazid
AE: adverse event
ART: antiretroviral therapy
ARV: antiretroviral
AUC: area under the plasma concentration-time curve
CD4 count: cluster of differentiation 4 count
CL: clearance
CONSORT: Consolidated Standards of Reporting Trials
DLT: dose-limiting toxicity
DSMB: Data and Safety Monitoring Board
MedDRA: Medical Dictionary for Regulatory Activities
PK: pharmacokinetics
TB: tuberculosis
TBTC: Tuberculosis Trials Consortium
TPT: tuberculosis preventive treatment
TST: tuberculin skin test
WHO: World Health Organization
